# Machine learning-based model for predicting tumor recurrence after interventional therapy in HBV-related hepatocellular carcinoma patients with low preoperative platelet-albumin-bilirubin score

**DOI:** 10.3389/fimmu.2024.1409443

**Published:** 2024-05-28

**Authors:** Qi Wang, Shugui Sheng, Yiqi Xiong, Ming Han, Ronghua Jin, Caixia Hu

**Affiliations:** ^1^ Interventional Therapy Center for Oncology, Beijing You’an Hospital, Capital Medical University, Beijing, China; ^2^ National Center for Infectious Diseases, Beijing Ditan Hospital, Capital Medical University, Beijing, China; ^3^ Changping Laboratory, Beijing, China

**Keywords:** hepatitis B virus (HBV), hepatocellular carcinoma (HCC), recurrence-free survival (RFS), extreme gradient boosting (Xgboost), random survival forest (RSF), nomogram

## Abstract

**Introduction:**

This study aimed to develop a prognostic nomogram for predicting the recurrence-free survival (RFS) of hepatitis B virus (HBV)-related hepatocellular carcinoma (HCC) patients with low preoperative platelet-albumin-bilirubin (PALBI) scores after transarterial chemoembolization (TACE) combined with local ablation treatment.

**Methods:**

We gathered clinical data from 632 HBV-related HCC patients who received the combination treatment at Beijing You’an Hospital, affiliated with Capital Medical University, from January 2014 to January 2020. The patients were divided into two groups based on their PALBI scores: low PALBI group (n=247) and high PALBI group (n=385). The low PALBI group was then divided into two cohorts: training cohort (n=172) and validation cohort (n=75). We utilized eXtreme Gradient Boosting (XGBoost), random survival forest (RSF), and multivariate Cox analysis to pinpoint the risk factors for RFS. Then, we developed a nomogram based on the screened factors and assessed its risk stratification capabilities and predictive performance.

**Results:**

The study finally identified age, aspartate aminotransferase (AST), and prothrombin time activity (PTA) as key predictors. The three variables were included to develop the nomogram for predicting the 1-, 3-, and 5-year RFS of HCC patients. We confirmed the nomogram’s ability to effectively discern high and low risk patients, as evidenced by Kaplan-Meier curves. We further corroborated the excellent discrimination, consistency, and clinical utility of the nomogram through assessments using the C-index, area under the curve (AUC), calibration curve, and decision curve analysis (DCA).

**Conclusion:**

Our study successfully constructed a robust nomogram, effectively predicting 1-, 3-, and 5-year RFS for HBV-related HCC patients with low preoperative PALBI scores after TACE combined with local ablation therapy.

## Introduction

Liver cancer, with hepatocellular carcinoma (HCC) as its main subtype, ranks as the third leading cause of cancer-related deaths globally (1). In developing regions such as China and Africa, chronic hepatitis B virus (HBV) infection serves as the predominant factor contributing to HCC (2, 3). Despite the considerable progress in treatment modalities witnessed in recent years, the prognosis of HCC patients remains unsatisfactory, primarily due to its high recurrence rates (4, 5). The prognosis of HCC is often closely related to liver function, so it is crucial to conduct a thorough liver function assessment before treatment (6–8). The traditional method for clinically assessing liver function is the Child-Pugh class, which includes five indicators: albumin, bilirubin, prothrombin time, ascites, and hepatic encephalopathy (9). However, the grading values are solely defined by clinical experience, and ascites and hepatic encephalopathy are susceptible to subjective factors, making it challenging to objectively and accurately evaluate liver function. In 2015, the albumin-bilirubin (ALBI) score, solely based on laboratory parameters, was introduced, and numerous clinical studies have validated its accuracy in predicting prognosis (10–12). However, as HCC often occurs in patients with cirrhosis, the degree of portal hypertension is a crucial prognostic factor. The ALBI score fails to assess the extent of portal hypertension. The platelet-albumin-bilirubin (PALBI) score, incorporating platelet count as a marker for portal hypertension, has been confirmed as a more ideal method for assessing liver function (13–15).

PALBI score categorizes HCC patients into three grades, with higher grades indicating poorer liver function and worse prognosis. Our team has also confirmed that there was a significant statistical difference in prognosis between HBV-related HCC patients with low and high PALBI grades. Specifically, patients with high PALBI scores had a more unfavorable prognosis compared to patients with low PALBI scores. However, although there were differences between the two, the prognosis of low PALBI patients was not very optimistic and their recurrence-free survival (RFS) was not much better than that of high PALBI patients. This finding suggests that for patients with low PALBI scores, caution should still be exercised in managing their prognosis, to fully understand and address potential recurrence risks. Unfortunately, there is currently no specialized prognostic prediction model for low PALBI patients.

Therefore, in this study, we not only investigated the impact of high and low PALBI scores on HBV-related HCC prognosis, but also committed to developing an RFS prediction nomogram particularly for low PALBI patients. In the process of establishing the nomogram, we integrated clinical characteristics, biochemical indicators, and tumor biology features of patients with low PALBI scores, employing advanced machine learning algorithms and statistical methods to systematically analyze and select factors influencing tumor recurrence. The goal is to construct an accurate, reliable, and clinically practical predictive model to assist clinicians in better assessing the recurrence risk of patients, providing essential reference for further optimization of treatment plans.

## Materials and methods

### Patients enrolled

In this retrospective study, data from 632 patients diagnosed with HBV-related HCC and treated with a combination of transarterial chemoembolization (TACE) and ablation treatment at Beijing You’an Hospital, affiliated with Capital Medical University, were reviewed, from January 2014 to January 2020. All participants enrolled presented with a confirmed diagnosis of HCC according to the criteria stipulated by the American Association for the Study of Liver Disease (AASLD). The combination treatment was undertaken by physicians possessing a minimum of 5 years of expertise in the field. TACE was initially employed to target the cancerous lesions, delivering chemotherapy drugs and embolic agents directly to the tumor-feeding blood vessels. Following the TACE procedure, local ablation therapy was performed within a time frame of 1 to 2 weeks.

**Figure 1:**
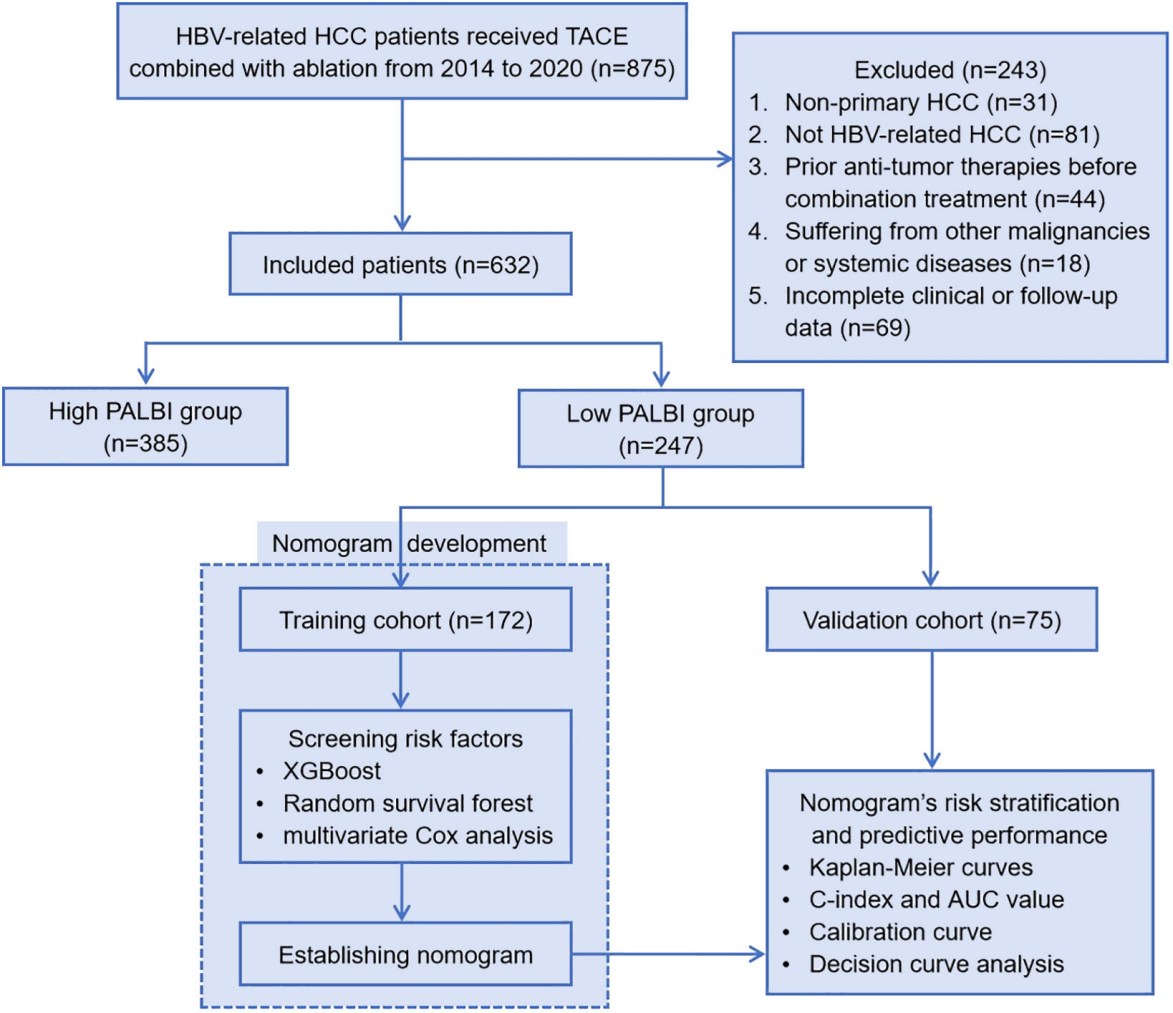
Patient enrollment flowchart. HBV, hepatitis B virus; HCC, hepatocellular carcinoma; TACE, transcatheter arterial chemoembolization; PALBI, platelet-albumin-bilirubin; XGBoost, eXtreme Gradient Boosting; C-index, Harrell’s concordance index; AUC, area under the curve.

Here are the inclusion and exclusion criteria for the study. Inclusion criteria: (1) Patients diagnosed with hepatitis B virus (HBV)-related primary HCC; (2) Received the combination of transarterial chemoembolization (TACE) and ablation treatment; (3) Complete clinical and follow-up data. Exclusion criteria: (1) Non-primary HCC; (2) Not HBV-related HCC; (3) Received other anti-tumor therapies before the combination treatment; (4) Suffering from other malignancies or systemic diseases; (5) Incomplete clinical or follow-up data. **Figure 1** illustrates the flowchart outlining the process of selecting qualified patients.

The study has obtained approval from the ethics committee of Beijing You’an Hospital, affiliated with Capital Medical University. The committee conducted a thorough review of the research protocol to ensure that the study adheres to ethical guidelines and regulatory requirements. Given the retrospective nature of the study, where data was collected from past medical records, and the stringent measures implemented to protect patient’s privacy, the need for obtaining informed consent from the subjects was deemed unnecessary.

### Data collection

The data collected in this study comprises general information, laboratory examination data, and pathological data. General information: age, gender, smoking history, drinking history, family history, antiviral history, hypertension, diabetes and so on. Laboratory examination data: white blood cell (WBC), red blood cell (RBC), lymphocyte (Lym), neutrophil (Neu), monocyte (Mon), eosinophils, basophils, platelet (PLT), hemoglobin (Hb), alanine aminotransferase (ALT), aspartate aminotransferase (AST), AST/ALT, gamma glutamyl transpeptidase (GGT), alkaline phosphatase (ALP), total protein (TP), albumin, globulin, prealbumin, total bilirubin (TBIL), direct bilirubin (DBIL), bile acid, uric acid, BUN, cholesterol, glucose, potassium, natrium, chlorine, creatinine, fibrinogen (Fib), thrombin time (TT), international normalized ratio (INR), activated partial thromboplastin time (APTT), activated partial thromboplastin time ratio (APTTR), prothrombin time (PT), prothrombin time activity (PTA), prothrombin time ratio (PTR) and so on. Pathological data: tumor size, tumor number, Barcelona Clinic Liver Cancer (BCLC) stage and Child-Pugh class.

### Patients follow-up

Within one month after the combination treatment, all patients were required to undergo a follow-up check at the outpatient department of our hospital. They were followed every 3 months starting from the 2nd month to 1 year and every six months thereafter. Tumor recurrence refers to the discovery of new lesions in the liver through imaging examinations such as contrast-enhanced computed tomography (CT) and magnetic resonance imaging (MRI) or confirmed by pathological evidence based on tumor biopsy. Recurrence-free survival (RFS) was calculated as the time interval from the date of undergoing treatment to the date of tumor recurrence or the last follow-up date, and was presented in months. The last follow-up date for this study was December 1, 2023 and the median follow-up time was 23.2 months.

### Statistical analysis

Categorical variables were expressed as numbers and proportions, and their comparison was executed through the Chi-square test. Continuous variables were represented as mean values accompanied by standard deviations, and their comparison was carried out using Student’s t-test or Mann-Whitney U test. The significance level was P=0.05, where P<0.05 indicated statistical difference. Platelet-albumin-bilirubin (PALBI) score was calculated as: 2.02 * log_10_ bilirubin - 0.37 * (log_10_ bilirubin)^2^ - 0.04 * albumin - 3.48 * log_10_ platelets + 1.01 * (log_10_ platelets)^2^, bilirubin was expressed in μmol/L, albumin in g/L and blood platelet count in 1000/μL. PALBI was divided into three grades, with higher grades indicating poorer liver function. PALBI grade 1: PALBI score≤ -2.53; PALBI grade 2: -2.53 < PALBI score ≤ -2.09; PALBI grade 3: PALBI score > -2.09. In this study, PALBI grade 1 was classified as low PALBI, while grade 2 and 3 were included as high PALBI. Survival analysis was conducted using Kaplan-Meier curves, and the log-rank test was utilized for data comparison. Subsequently, patients categorized under the low PALBI group were randomly split into training and validation cohorts in a 7:3 ratio. Then, we utilized a combination of advanced analytical techniques, including eXtreme Gradient Boosting (XGBoost), random survival forest (RSF), and multivariate Cox analysis, to delve into the factors influencing RFS following treatment for HBV-related HCC with low PALBI score. Based on the screened risk factors, we developed a nomogram to predict the 1-year, 3-year and 5-year RFS of low PALBI HBV-related HCC patients. Furthermore, using the scores generated by the nomogram, patients were effectively categorized into two distinct risk groups: low-risk and high-risk groups. The survival curves of the two groups were also compared by the log-rank test. Moreover, the discrimination ability, calibration, and clinical utility of the nomogram were then comprehensively evaluated to ensure its robustness and reliability.

## Results

### Comparison of RFS between high PALBI group and low PALBI group

**Figure 2:**
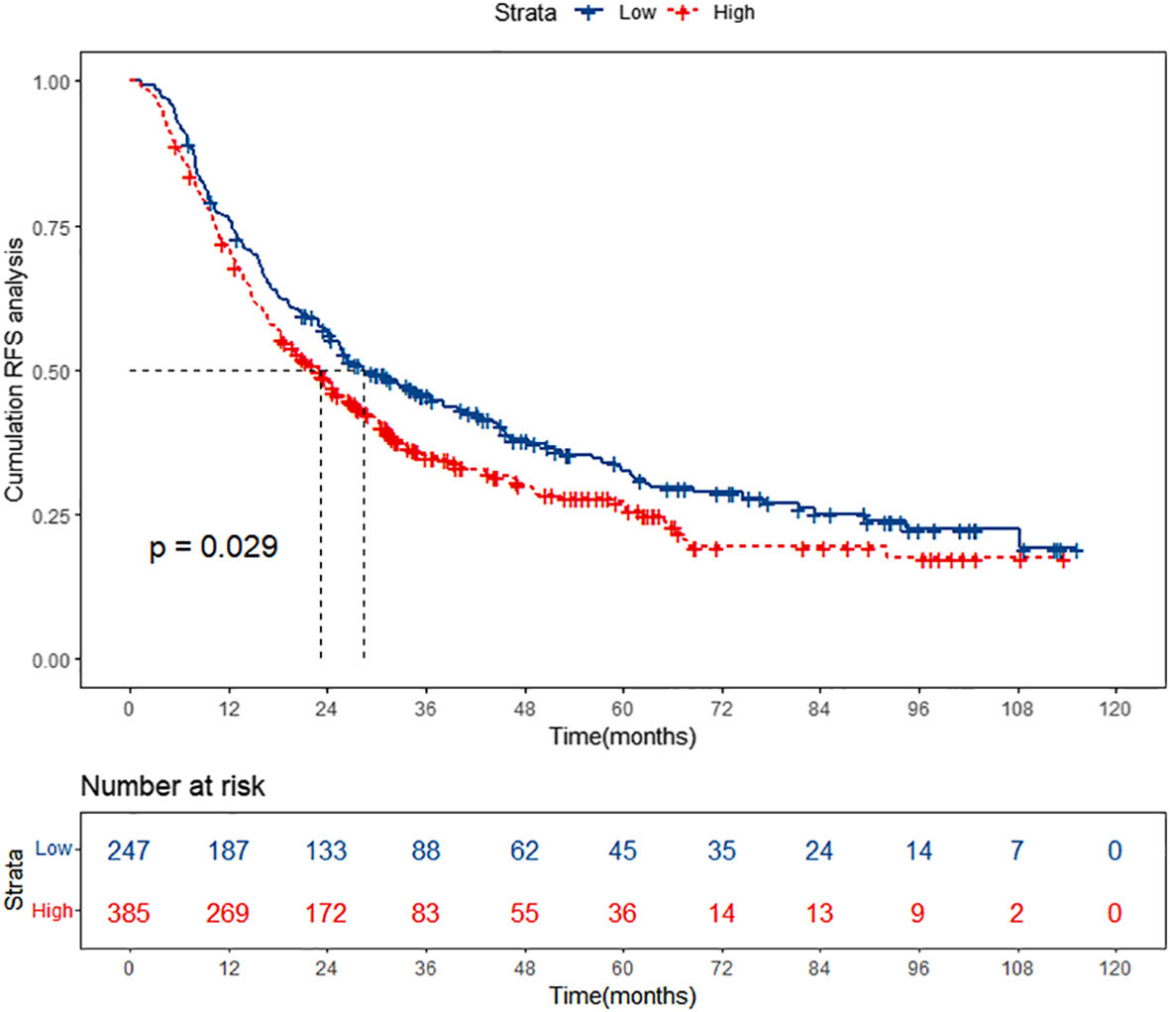
Kaplan-Meier curves of high PALBI group and low PALBI group. PALBI, platelet-albumin-bilirubin; RFS, recurrence-free survival.

A total of 632 patients diagnosed with HBV-related HCC who underwent TACE combined with ablation therapy were included in this retrospective study. The patients were stratified into two groups according to their preoperative PALBI scores: the low PALBI group comprised 247 individuals, while the high PALBI group consisted of 385 patients. It was confirmed that the high PALBI group exhibited higher recurrence rates compared to low PALBI group. However, delving deeper into the data, we unearthed a rather intriguing revelation: while there existed a disparity between these two groups, the RFS of patients with low PALBI scores did not demonstrate a substantial advantage over that of their high PALBI counterparts, as shown in **Figure 2**. It implies that despite falling into the low PALBI category, patients were not necessarily immune to recurrence risks and warrant meticulous attention in managing their prognosis. This revelation underscores the imperative of developing specialized prognostic prediction models tailored to this subgroup, enabling clinicians to accurately assess recurrence risks and implement targeted interventions that optimize patient outcomes.

### Baseline characteristics of the low PALBI patients

The group of 247 patients with low PALBI scores was divided into two cohorts, the training cohort (n=172) and the validation cohort (n=75), through random assignment, maintaining a ratio of 7 to 3. To ensure the validity of our analysis, a meticulous comparison of baseline characteristics between the training and validation cohorts was conducted. This comparative analysis aimed to ascertain whether there were any differences in the distribution of variables between the two cohorts. The results, as summarized in **Table 1**, unequivocally demonstrated that there existed no statistically significant difference (P > 0.05) in the baseline characteristics between the training and validation cohorts, indicating their suitability for further studies.

**Table 1:** Baseline characteristics of patients in training and validation cohorts.

Characteristic	Training cohort (N=172)	Validation cohort (N=75)	P value
Gender (male/female)	117 (68.0%)/55 (32.0%)	52 (69.3%)/23 (30.7%)	0.839
Child-Pugh class (A/B)	163 (94.8%)/9 (5.2%)	73 (97.3%)/2 (2.7%)	0.369
Tumor number (Single/multiple)	120 (69.8%)/52 (30.2%)	57 (76.0%)/18 (24.0%)	0.318
Tumor size (≤3cm/>3cm)	123 (71.5%)/49 (28.5%)	61 (81.3%)/14 (18.7%)	0.103
Age	55.58 ± 9.57	55.04 ± 9.55	0.686
Lym	1.37 ± 0.62	1.34 ± 0.58	0.738
PLT	124.87 ± 49.50	129.03 ± 48.91	0.543
AST	29.14 ± 13.56	26.99 ± 12.23	0.240
DBIL	3.85 ± 2.32	3.70 ± 1.72	0.616
Albumin	40.60 ± 4.24	40.57 ± 3.09	0.942
Globulin	26.73 ± 3.82	27.05 ± 4.26	0.551
GGT	52.06 ± 40.96	48.83 ± 33.83	0.550
Prealbumin	160.50 ± 50.72	168.54 ± 60.96	0.283
Bile acid	11.82 ± 11.77	10.79 ± 11.79	0.527
Creatinine	64.60 ± 16.25	89.35 ± 152.23	0.164
Uric acid	282.57 ± 81.69	305.07 ± 90.29	0.055
Glucose	8.52 ± 26.06	5.99 ± 2.13	0.404
Cholesterol	4.16 ± 2.53	3.90 ± 0.82	0.381
Potassium	3.99 ± 0.33	4.02 ± 0.40	0.505
PT	11.96 ± 1.02	11.81 ± 0.86	0.273
PTA	91.80 ± 12.32	93.15 ± 10.70	0.411
PTR	1.07 ± 0.12	1.04 ± 0.10	0.059
INR	1.06 ± 0.09	1.05 ± 0.08	0.322
Fib	2.86 ± 0.85	2.89 ± 0.79	0.797

Lym, lymphocyte; PLT, platelet; AST, aspartate aminotransferase; DBIL, direct bilirubin; GGT, gamma glutamyl transpeptidase; PT, prothrombin time; PTA, prothrombin time activity; PTR, prothrombin time ratio; INR, international normalized ratio; Fib, fibrinogen.

### Screening risk factors associated with RFS through XGBoost, RSF, and multivariate Cox analysis

**Figure 3:**
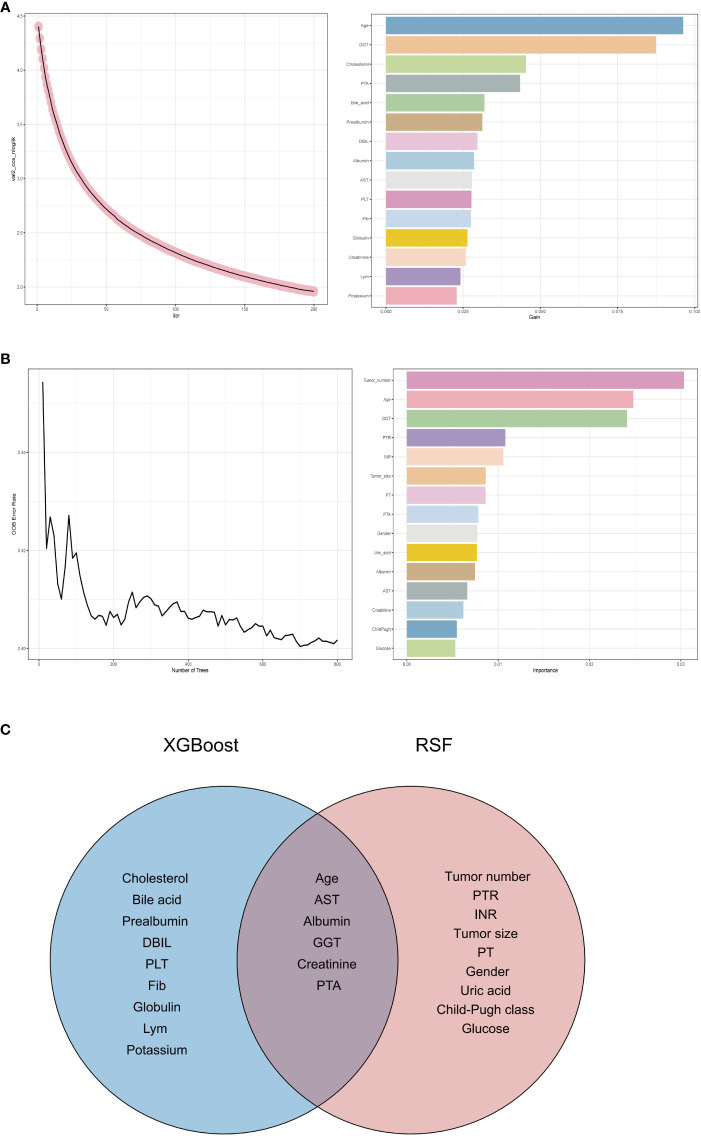
Variable selection process. **(A)** Variable selection for RFS using eXtreme Gradient Boosting. **(B)** Variable selection for RFS using random survival forest. **(C)** Venn diagram showing the intersection of eXtreme Gradient Boosting and random survival forest. RFS, recurrence-free survival; GGT, gamma glutamyl transpeptidase; PTA, prothrombin time activity; DBIL, direct bilirubin; AST, aspartate aminotransferase; PLT, platelet; Fib, fibrinogen; Lym, lymphocyte; PTR, prothrombin time ratio; INR, international normalized ratio; PT, prothrombin time; PTA, prothrombin time activity.

In this study aimed at screening the factors influencing RFS, a multifaceted approach combining machine learning techniques and traditional statistical analysis was employed. At first, we utilized XGBoost, a powerful machine learning algorithm known for its effectiveness in handling complex datasets, to screen for relevant variables affecting RFS (16, 17). XGBoost is particularly adept at capturing nonlinear relationships and interactions within the data, making it suitable for identifying intricate patterns that might influence RFS outcomes. **Figure 3A** shows the results of XGBoost and presents the top 15 important variables, including age, GGT, cholesterol, PTA, bile acid, prealbumin, DBIL, albumin, AST, PLT, Fib, globulin, creatinine, Lym and potassium.

We then employed another machine learning technique - random survival forest (RSF), to scrutinize variables affecting RFS. **Figure 3B** depicts the outcomes of the RSF analysis, showcasing the top 15 significant variables, which encompass tumor number, age, GGT, PTR, INR, tumor size, PT, PTA, gender, uric acid, albumin, AST, creatinine, Child-Pugh class and glucose.

Subsequently, we took the intersection of the top 15 important variables for each of these two methods, as shown in **Figure 3C**. It can be seen that age, AST, albumin, GGT, creatinine and PTA are their shared variables. This strategic approach enabled us to pinpoint variables that demonstrated consistent importance across different analytical frameworks. By focusing on the shared variables identified through this process, we ensured a more robust and reliable selection of predictors for further analysis.

Following that, we engaged in a rigorous screening process using multivariate Cox regression analysis. This statistical technique allowed us to assess the significance of each variable while controlling for potential confounding factors. Variables with a significance level below 0.05 were deemed statistically significant and identified as independent risk factors influencing RFS outcomes. **Table 2** shows the results of multivariate Cox analysis. We finally identified age, AST, and PTA as independent risk factors. This meticulous approach ensured that only the most relevant and influential variables were considered in our analysis.

**Table 2:** Multivariate Cox regression analysis results.

Variable	Hazard Ratio (95%CI)	P value
**Age**	**1.042 (1.021–1.064)**	**<0.001**
**AST**	**1.515 (1.098–1.931)**	**0.017**
Albumin	0.99 (0.944–1.037)	0.663
GGT	1.003 (0.999–1.008)	0.157
Creatinine	1.011 (0.998–1.023)	0.103
**PTA**	**0.98 (0.964–0.997)**	**0.02**

AST, aspartate aminotransferase; GGT, gamma glutamyl transpeptidase; PTA, prothrombin time activity. The bold values suggest the variables with P<0.05.

### Nomogram developed for RFS prediction using the screened factors

**Figure 4:**
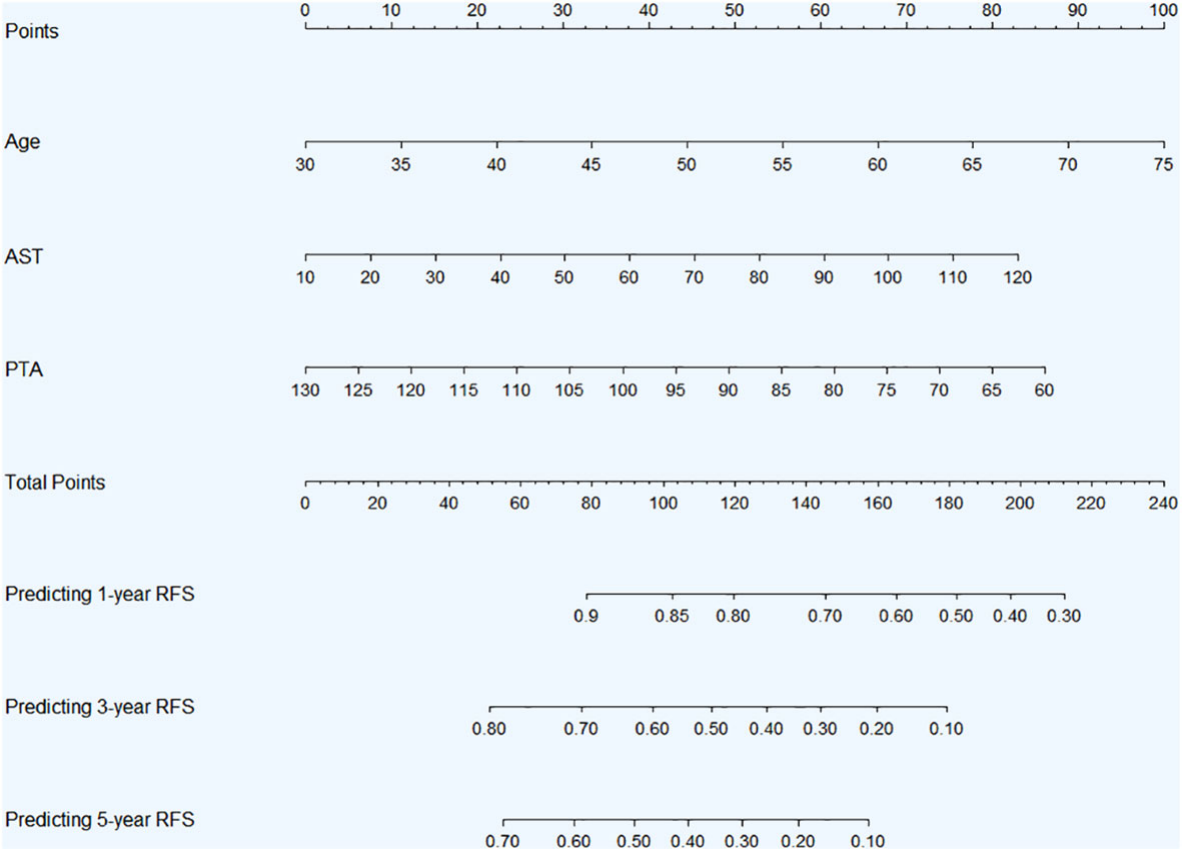
Nomogram for 1-, 3-, and 5-year RFS prediction in HBV-related HCC patients with low preoperative PALBI score after TACE combined with ablation therapy. **Abbreviations:** RFS, recurrence-free survival; HBV, hepatitis B virus; HCC, hepatocellular carcinoma; PALBI, platelet-albumin-bilirubin; TACE, transcatheter arterial chemoembolization; AST, aspartate aminotransferase; PTA, prothrombin time activity.

Through the above rigorous statistical analysis, factors (age, AST and PTA) significantly associated with RFS were identified and incorporated into the nomogram (**Figure 4**). This nomogram serves as a practical aid in clinical settings, providing a visual representation of the identified factors’ contributions to the overall risk assessment for RFS. The basic principle of the nomogram, simply put, is to assign scores to each value of each influencing factor based on the contribution (i.e., the values of regression coefficients) of these factors to the RFS in the multivariate Cox regression model. These scores are then summed up to obtain a total score, which is used to predict the 1-year, 3-year and 5-year RFS probabilities of patients.

### The Kaplan-Meier curves of low and high-risk groups stratified by nomogram scores

**Figure 5:**
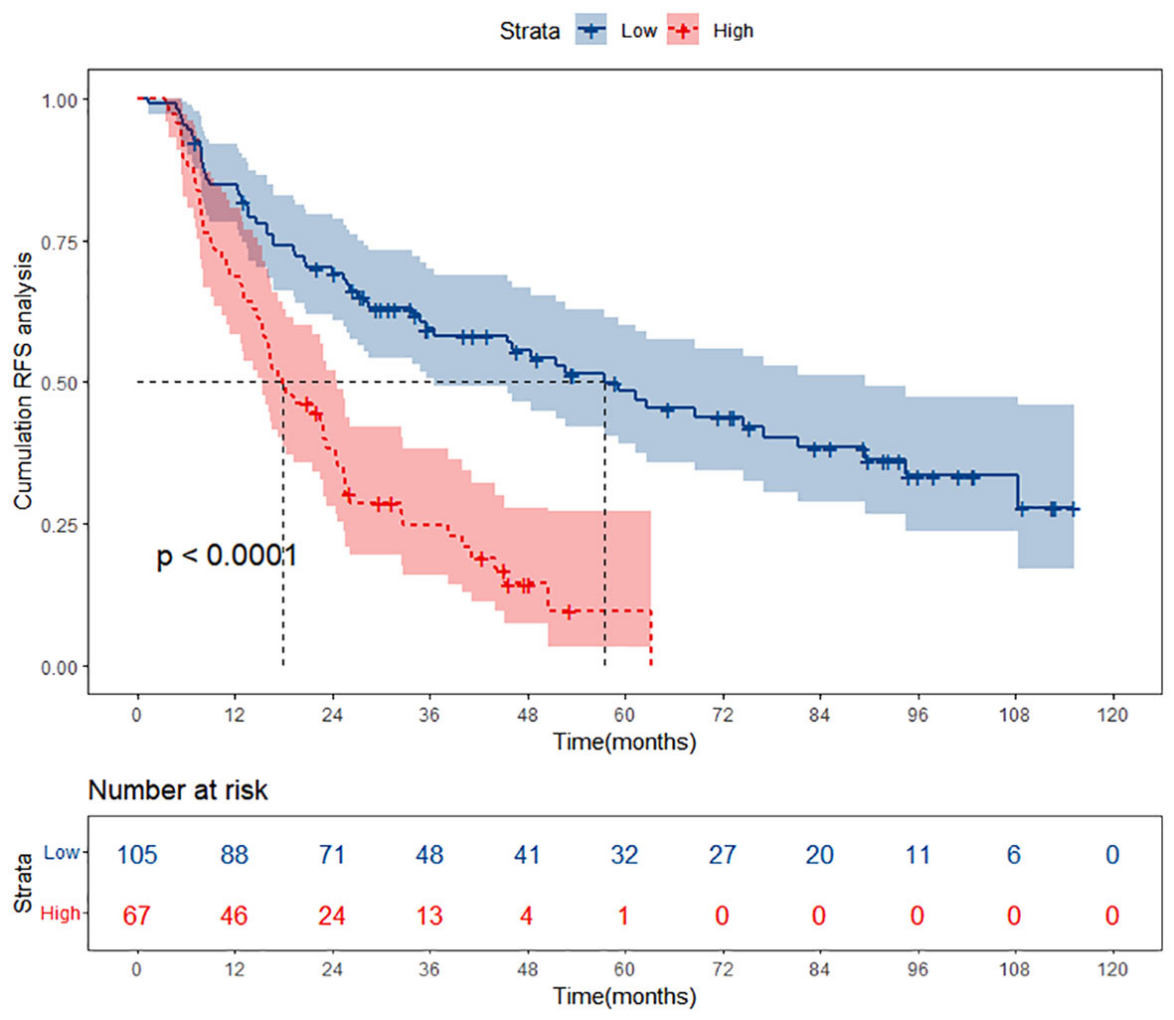
Kaplan-Meier curves of different risk groups stratified by nomogram-derived points in the training cohort. RFS, recurrence-free survival.

In both the training and validation cohorts, patients were categorized into two risk groups—low-risk and high-risk—based on the scores generated by the nomogram. Subsequently, Kaplan-Meier curves were plotted for both the low and high-risk groups to assess their RFS outcomes. In the training cohort, the Kaplan-Meier curves revealed a clear demarcation between the low-risk and high-risk groups (**Figure 5**). Patients classified as low-risk exhibited a more favorable prognosis, with a higher likelihood of survival over the follow-up period. Conversely, those categorized as high-risk faced a greater risk of recurrence, as evidenced by a steeper decline in survival probability. Similarly, the validation cohort exhibited comparable trends, further validating the prognostic utility of the nomogram-based risk stratification (**Supplementary Figure S1**).

### Evaluating the performance of the nomogram

**Figure 6:**
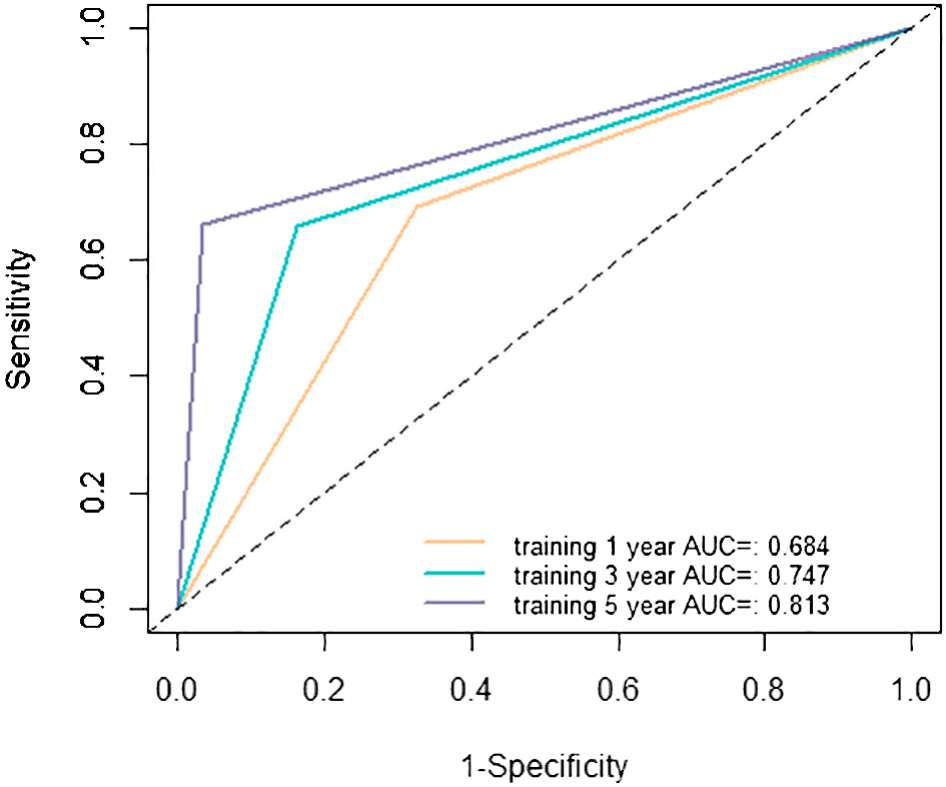
Receiver operating characteristic (ROC) curves in the training cohort. AUC, area under the curve.

**Figure 7:**
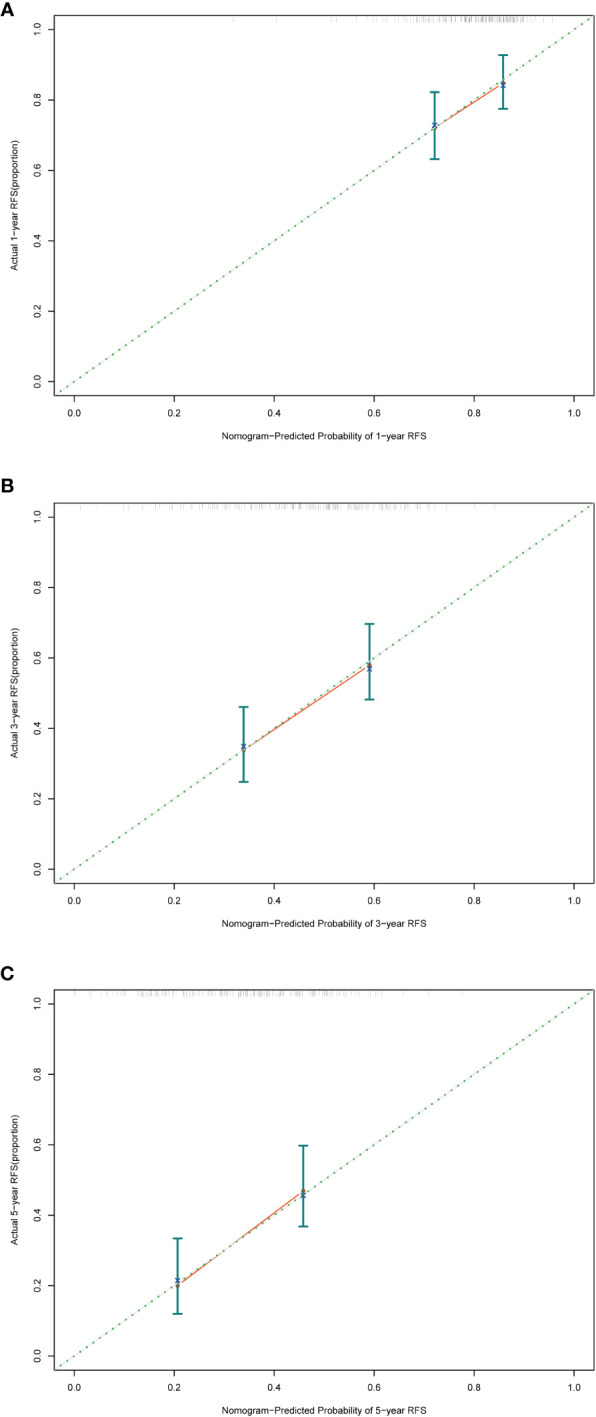
Calibration curves in the training cohort. **(A)** Calibration curve of 1-year RFS. **(B)** Calibration curve of 3-year RFS. **(C)** Calibration curve of 5-year RFS. RFS, recurrence-free survival.

**Figure 8:**
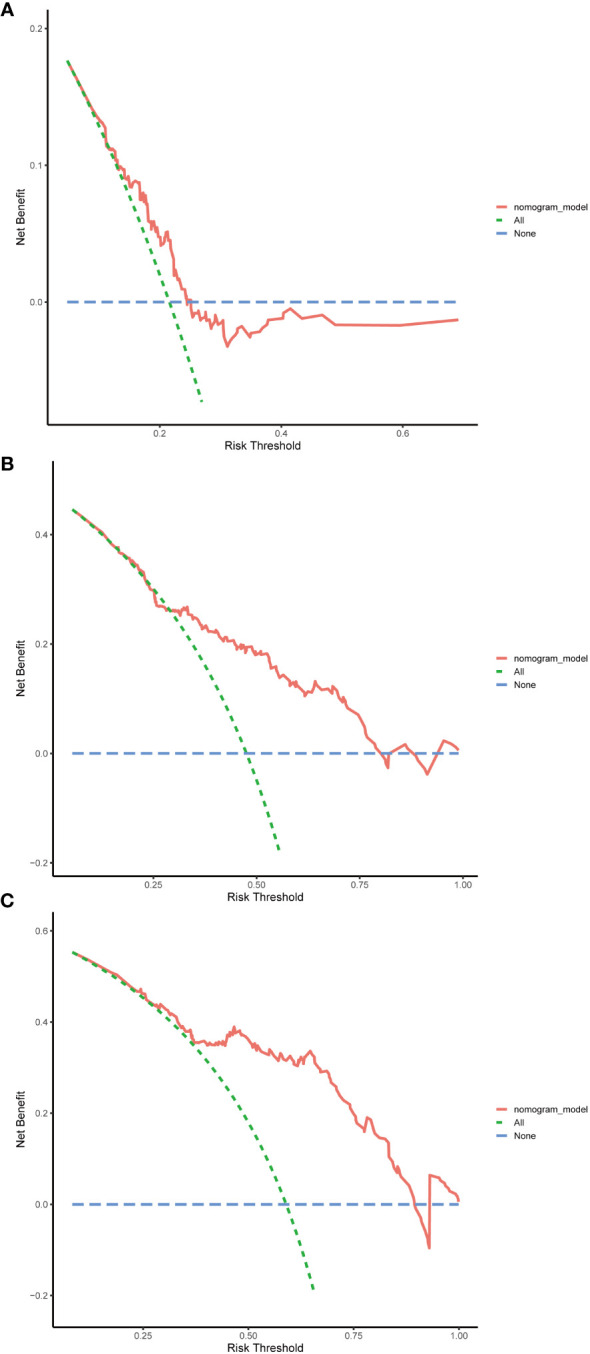
Decision curve analysis (DCA) in the training cohort. **(A)** DCA curve of 1-year RFS. **(B)** DCA curve of 3-year RFS. **(C)** DCA curve of 5-year RFS. RFS, recurrence-free survival.

The evaluation of the nomogram’s performance is crucial for assessing its accuracy and reliability in predicting outcomes. Several metrics can be employed to gauge its effectiveness. Harrell’s concordance index (C-index) and Area Under the Curve (AUC) were used to evaluate its discrimination ability. Calibration curve was used to assess the agreement between predicted probabilities generated by the nomogram and observed outcomes. Decision curve analysis (DCA) was employed to evaluate its clinical utility. In the training cohort, we found that the nomogram exhibited strong discrimination ability. Its C-index was 0.689 (95% CI: 0.64–0.738) and its AUC values for 1-year, 3-year and 5-year RFS were 0.684, 0.747 and 0.813, respectively (**Figure 6**). The 1-year, 3-year and 5-year calibration curves closely approximated the ideal diagonal line, indicating consistent predictions with actual observations (**Figure 7**). The results of 1-year, 3-year and 5-year DCA curves demonstrated significant utility and clinical relevance (**Figure 8**). The analysis indicated that the nomogram’s predictions offer substantial net benefit across a range of thresholds, highlighting its potential for guiding clinical decision-making effectively. In the validation cohort, the nomogram also demonstrated excellent predictive efficacy, receiving favorable evaluations across these metrics. The C-index of the nomogram in the validation cohort was 0.642 (95% CI: 0.569–0.714). Its 1-year, 3-year and 5-year AUC values were 0.689, 0.716 and 0.747, respectively (**Supplementary Figure S2**). The calibration (**Supplementary Figure S3**) and DCA (**Supplementary Figure S4**) curves both showcased outstanding performance, affirming the model’s accuracy and significant clinical utility.

## Discussion

The absence of a specialized prognostic prediction model for low PALBI HBV-related HCC patients after receiving TACE combined with ablation therapy poses a considerable challenge in clinical management. To address this gap, our team embarked on comprehensive research aimed at developing a tailored prognostic nomogram specifically designed for this patient subgroup. Compared with past HCC prognostic models, this study has several significant innovations. Our study distinctively focuses on low PALBI score patients, a subgroup typically overlooked in previous research. Instead of treating all patients uniformly, we meticulously explore the unique characteristics and prognostic factors of these individuals, providing more targeted guidance for personalized treatment strategies. Furthermore, we employed cutting-edge machine learning algorithms to develop our prognostic prediction model. Unlike conventional statistical approaches, these algorithms excel in uncovering intricate patterns within data, thereby enhancing the accuracy and stability of our predictions. Through utilizing these advanced technologies, our nomogram offers a more precise recurrence evaluation for low PALBI HBV-related HCC patients.

The nomogram of this study included age, AST, and PTA as predictive indicators. We found that with increasing age, patients with HCC are at a higher risk of recurrence. As age increases, the functionality of the human immune system gradually declines, potentially leading to weakened immune surveillance against tumors and consequently HCC development (18). Furthermore, age-related comorbidities may also affect the prognosis of HCC patients. However, not all studies uniformly support the association between age and HCC development. Some studies have found that, under the same treatment regimens, there is no significant difference in the prognosis of HCC between elderly patients and younger patients (19–22). AST also plays a crucial role in the recurrence of HCC in this study. The elevated serum AST in patients is positively correlated with the degree of liver cell damage. Some scholars have found that persistently elevated AST levels may be associated with poorer survival rates and disease progression (23–26). Therefore, regular monitoring of AST levels may help predict the prognosis of HCC patients. PTA is an indicator used to evaluate coagulation function (27). PTA (%) = (normal person’s prothrombin time - 8.7)/(patient’s prothrombin time - 8.7) * 100. Prothrombin is one of the vitamin K-dependent coagulation factors synthesized by the liver, so it is essential to measure its activity in liver function tests. It is a sensitive indicator for assessing the severity of hepatocyte necrosis and prognosis. When the liver is damaged or impaired, as in conditions like hepatitis, cirrhosis and HCC, the synthesis of prothrombin is compromised, leading to prolonged patient’s prothrombin time and decreased PTA levels. Many studies have pointed out that prolonged prothrombin time is associated with higher morbidity and mortality in patients (28–30), while few studies have focused on the more precise expression of prothrombin time, PTA. This suggests that by monitoring patients’ preoperative PTA levels, it is possible to assess treatment risks in advance and take corresponding preventive measures to improve the success rate of treatment and patient survival.

The future of HCC prognosis models is poised at an exciting convergence of biotechnology, data science, and patient-centered care, heralding a new era of precision oncology. Advances in genomic sequencing and bioinformatics are enabling more nuanced understandings of HCC’s molecular and genetic underpinnings, driving the development of prognosis models that incorporate multi-omics data—genomic, transcriptomic, proteomic, and metabolomic—to offer predictions tailored to the individual genetic and molecular profile of the patient (28, 31–34). This multi-omics approach promises to refine risk stratification, guide therapy selection, and predict therapeutic responses with unprecedented accuracy. Parallelly, artificial intelligence (AI), deep learning (DL) and machine learning (ML) technologies are transforming the landscape of prognostic modeling (35–38). By harnessing vast datasets of patient records, imaging studies, and omics data, AI algorithms can identify patterns and prognostic markers that are imperceptible to the human eye. These AI-driven models are expected to improve continuously through iterative learning, becoming more precise and reliable as they are fed more data. The integration of AI with electronic health records and digital health technologies will further personalize prognosis and treatment plans, incorporating real-time health data from wearable devices and patient-reported outcomes to dynamically adjust prognostic predictions and therapeutic recommendations. Furthermore, the integration of liquid biopsy technologies into HCC prognosis models is a trend with significant potential. By enabling the non-invasive detection of circulating tumor DNA (ctDNA) and circulating tumor cells, liquid biopsies offer a means to monitor tumor burden, detect minimal residual disease, and identify emerging resistance mutations in real time (39–43). This technology could revolutionize HCC management, allowing for earlier intervention, the adaptation of treatment strategies in response to tumor evolution, and improved prognostic accuracy.

Therefore, future models are likely to incorporate patient preferences, quality of life measures, and socio-economic factors alongside traditional clinical and molecular data, facilitating shared decision-making and tailored treatment plans that align with patient values and life goals. In addition, in future research, we will seek to cooperate with other medical institutions to conduct multi-center studies to further verify the effectiveness of the nomogram we developed.

## Conclusion

Our study successfully constructed a robust nomogram, effectively predicting 1-, 3-, and 5-year RFS for HBV-related HCC patients with low preoperative PALBI scores after TACE combined with local ablation therapy.

## Data availability statement

The original contributions presented in the study are included in the article/**Supplementary Material**. Further inquiries can be directed to the corresponding authors.

## Ethics statement

The studies involving humans were approved by Ethics Committee of Beijing You’an Hospital, affiliated with Capital Medical University. The studies were conducted in accordance with the local legislation and institutional requirements. The ethics committee/institutional review board waived the requirement of written informed consent for participation from the participants or the participants’ legal guardians/next of kin because Given the retrospective nature of the study, where data was collected from past medical records, and the stringent measures implemented to protect patient’s privacy, the need for obtaining informed consent from the subjects was deemed unnecessary.

## Author contributions

QW: Writing – review & editing, Methodology, Data curation, Formal Analysis, Writing – original draft. SS: Formal Analysis, Methodology, Writing – original draft, Writing – review & editing. YX: Writing – review & editing, Data curation, Validation. MH: Writing – review & editing, Conceptualization, Methodology, Project administration. RJ: Conceptualization, Methodology, Project administration, Writing – review & editing. CH: Conceptualization, Funding acquisition, Supervision, Writing – review & editing.

## Supplementary material

Supplementary Figure 1 Kaplan-Meier curves of different risk groups stratified by nomogram-derived points in the validation cohort. RFS, recurrence-free survival.

Supplementary Figure 2 Receiver operating characteristic (ROC) curves in the validation cohort. AUC, area under the curve.

Supplementary Figure 3 Calibration curves in the validation cohort. **(A)** Calibration curve of 1-year RFS. **(B)** Calibration curve of 3-year RFS. **(C)** Calibration curve of 5-year RFS. RFS, recurrence-free survival.

Supplementary Figure 4 Decision curve analysis (DCA) in the validation cohort. **(A)** DCA curve of 1-year RFS. **(B)** DCA curve of 3-year RFS. **(C)** DCA curve of 5-year RFS. RFS, recurrence-free survival.
