# Supplementary figures and images for: Machine learning-based model for predicting tumor recurrence after interventional therapy in HBV-related hepatocellular carcinoma patients with low preoperative platelet-albumin-bilirubin score
Source: Front Immunol. 2024 May 28;15:1409443. doi: 10.3389/fimmu.2024.1409443 (PMC11165108; doi:10.3389/fimmu.2024.1409443)

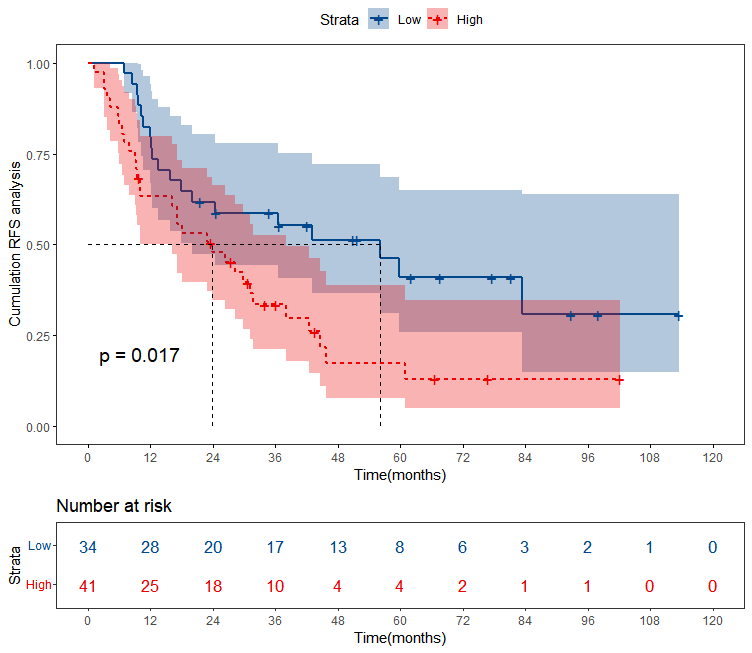

Supplement: Supplementary Figure 1 — Kaplan-Meier curves of different risk groups stratified by nomogram-derived points in the validation cohort. RFS, recurrence-free survival. [file Image_1.tiff]

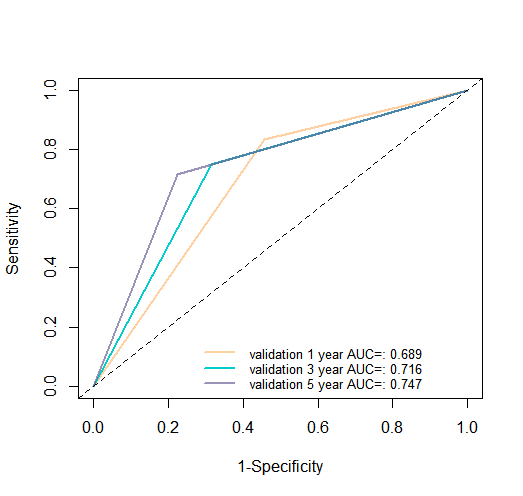

Supplement: Supplementary Figure 2 — Receiver operating characteristic (ROC) curves in the validation cohort. AUC, area under the curve. [file Image_2.tiff]

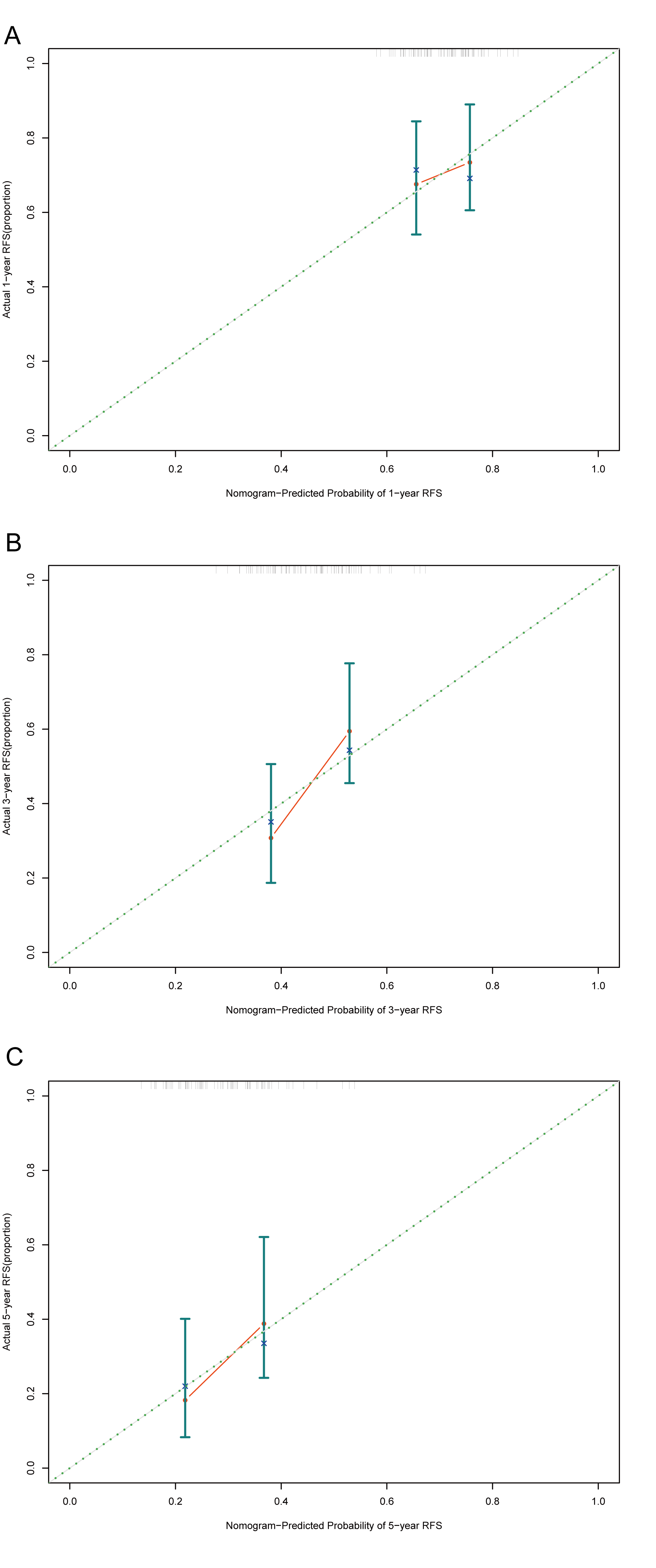

Supplement: Supplementary Figure 3 — Calibration curves in the validation cohort. (A) Calibration curve of 1-year RFS. (B) Calibration curve of 3-year RFS. (C) Calibration curve of 5-year RFS. RFS, recurrence-free survival. [file Image_3.tif]

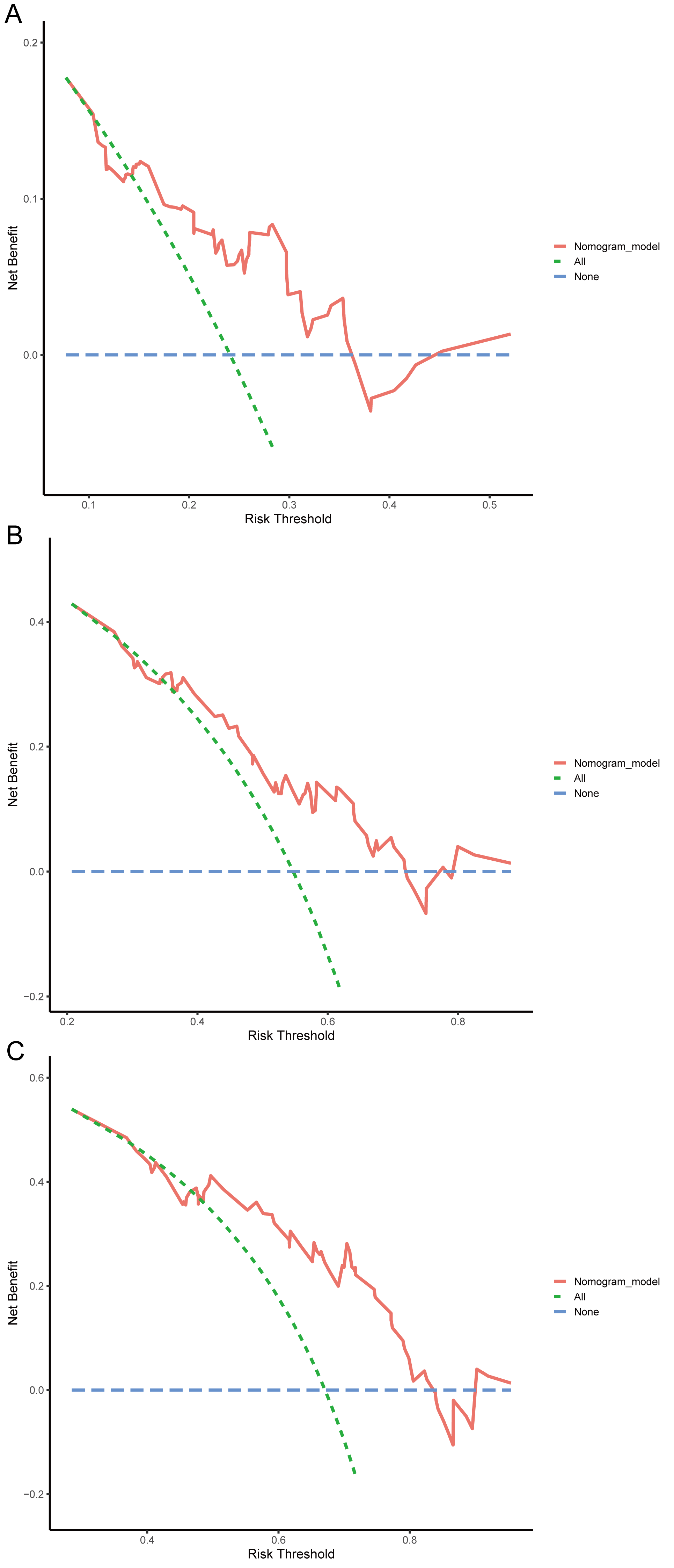

Supplement: Supplementary Figure 4 — Decision curve analysis (DCA) in the validation cohort. (A) DCA curve of 1-year RFS. (B) DCA curve of 3-year RFS. (C) DCA curve of 5-year RFS. RFS, recurrence-free survival. [file Image_4.tif]
